# A Multitask-Aided Transfer Learning-Based Diagnostic Framework for Bearings under Inconsistent Working Conditions

**DOI:** 10.3390/s20247205

**Published:** 2020-12-16

**Authors:** Md Junayed Hasan, Muhammad Sohaib, Jong-Myon Kim

**Affiliations:** 1 School of Computer Engineering and Information Technology, University of Ulsan, Ulsan 44610, Korea; junhasan@gmail.com; 2 Department of Computer Science, Lahore Garrison University, Lahore 54000, Pakistan; md.sohaibdurrani@gmail.com

**Keywords:** bearing, bispectrum, convolution neural network, fault diagnosis, multitask learning, transfer learning

## Abstract

Rolling element bearings are a vital part of rotating machines and their sudden failure can result in huge economic losses as well as physical causalities. Popular bearing fault diagnosis techniques include statistical feature analysis of time, frequency, or time-frequency domain data. These engineered features are susceptible to variations under inconsistent machine operation due to the non-stationary, non-linear, and complex nature of the recorded vibration signals. To address these issues, numerous deep learning-based frameworks have been proposed in the literature. However, the logical reasoning behind crack severities and the longer training times needed to identify multiple health characteristics at the same time still pose challenges. Therefore, in this work, a diagnosis framework is proposed that uses higher-order spectral analysis and multitask learning (MTL), while also incorporating transfer learning (TL). The idea is to first preprocess the vibration signals recorded from a bearing to look for distinct patterns for a given fault type under inconsistent working conditions, e.g., variable motor speeds and loads, multiple crack severities, compound faults, and ample noise. Later, these bispectra are provided as an input to the proposed MTL-based convolutional neural network (CNN) to identify the speed and the health conditions, simultaneously. Finally, the TL-based approach is adopted to identify bearing faults in the presence of multiple crack severities. The proposed diagnostic framework is evaluated on several datasets and the experimental results are compared with several state-of-the-art diagnostic techniques to validate the superiority of the proposed model under inconsistent working conditions.

## 1. Introduction

Rotating machinery is a prevailing component that plays an increasingly significant role in modern industries [1,2]. Due to their regular usage, these machines are subject to wear and tear processes, and it is necessary to perform predictive maintenance of these machines [3]. Rolling element bearings are vital components of rotating machinery. Harsh working environments, variable working conditions and changing load conditions are a few of the factors that can lead to the failure of a bearing. Thus, these components are often the primary reasons for the sudden failure of rotating machinery [1], which can create huge economic losses and personnel casualties [4,5,6,7]. Over the past few decades, industries have recognized the significance of developing reasonable and robust data-driven condition monitoring and fault diagnosis techniques to mitigate such problems [8]. Data-driven condition monitoring and fault diagnosis of a bearing normally consist of data acquisition from the bearing, signal processing and data classification steps. However, due to several important factors, e.g., friction, clearance, and variable working conditions, the acquired vibration signals from these rolling bearings are non-linear and non-stationary, which makes extracting fault feature information a difficult task [3,9,10,11,12,13,14]. Specifically, when using popular feature extraction methods that analyze features from the time domain, frequency domain, or time-frequency domain, it is very difficult to identify the fault characteristics under variable working conditions [15,16,17,18,19,20]. Therefore, research on new and effective methods for the condition monitoring of rolling element bearings has become a challenging and valuable task [21,22,23,24].

Recently, vibration signal analysis has become a standard for creating the diagnostic framework for rolling element bearings [25,26]. Many efforts have been conducted to identify the fault characteristics to create a generalized diagnostic framework by analyzing the vibration signals. For instance, Zheng et al. [27] introduced a multiscale fuzzy entropy-based architecture to measure the complexity of the time series using a combination of the Laplacian score (LS) and a variable predictive model to develop a bearing fault diagnostic framework. Similarly, in [28], Ali et al. proposed a model for bearing fault diagnosis based on statistical feature extraction from empirical mode decomposed signals and the energy and entropy values of the signals with an artificial neural network (ANN). Following a similar trend, Zhao et al. [29] performed bearing fault diagnosis using wavelet packet decomposition (WPD)–based, multi-scale permutation entropy with a hidden Markov model (HMM). Further, Shao et al. [30] proposed a diagnostic framework by using fast Fourier transformation (FFT) with a deep Boltzmann machine (DBM). By considering spectrum features from sliding windows, Wang et al. [31] developed a deep belief network (DBN)-based diagnostic model. These studies mainly extract fault signatures by analyzing the time-domain or frequency-domain and use deep learning algorithms to diagnose the health types of different mechanical machines. However, when using only the time or frequency domain, it is very difficult to capture the changing nature of frequency over time for non-stationary and non-linear signals [1]. Additionally, due to the disparity in the signal amplitude and frequency for several inconsistent working conditions, e.g., variable loads, speeds, different crack severities, and compound faults, these established approaches have failed to generalize the health characteristics. To solve these problems, several time-frequency-based analysis techniques have also been proposed. In [32], Wang et al. designed a wavelet-based time-frequency analysis with a CNN. In [33], a bearing fault diagnosis mechanism was proposed using short-time Fourier transform (STFT)-based time-frequency analysis with a CNN. These methods are mainly based on the analysis of one-dimensional acceleration signals through a deep learning algorithm for automatic feature extraction [34]. Nevertheless, despite a profound impact on the diagnostic framework [35], these types of methods can miss important information [34]. For instance, every so often, signal processing methods are difficult to implement because proper domain expertise is needed. Moreover, a particular signal analysis technique may only apply to a certain problem set; hence, the generalization capability of a developed fault diagnosis approach under various working conditions is compromised [36]. Fortunately, these types of automatic diagnostic approaches prove the drawbacks of handcrafted feature-based methods [37], which were popular for a certain period of time. Thus, as a newly established research direction, numerous studies have been conducted to improve deep learning-based automatic feature extraction for bearing fault diagnosis. The main goal of these types of domain-dependent automatic diagnostic frameworks is to develop a powerful feature extractor that can grab distinguishable features from input data. In [38], Jia et al. introduced neuron activation maximization to improve diagnostic deep algorithms for imbalanced data. Similarly, in [39], Liu et al. introduced a dislocated layer into the CNN architecture to boost the one-dimensional feature analysis performance. However, these methods have two major drawbacks: (a) the lack of proper health state information that is applicable in cross-domain fault diagnosis and (b) weak generalization capabilities because these approaches cannot provide satisfactory results for the fault diagnosis of bearings under inconsistent working conditions (e.g., variable motor speed, variable motor load, multiple crack severities, and compound fault types).

In this study, an automatic bearing diagnostic framework has been developed that utilizes higher-order spectral analysis of the vibration signal and multitask learning (MTL). The proposed model can identify bearing health conditions under inconsistent working conditions, e.g., in the presence of multiple fault severities, noisy conditions, compound faults, and variable motor speed, simultaneously. The higher-order spectral analysis projects the input signal on to a two-dimensional frequency space that adequately describes the health state of a bearing. In this way, it reduces the possibilities of deceptive information obtained during the analysis of non-stationary and non-linear vibration signals [1,40]. Subsequently, for fault identification and classification, a multitask learning (MTL)-based deep architecture is proposed. This helps the framework learn different tasks collectively. MTL allocates one shared model instead of using a separate model for different tasks, which helps reduce the storage space and training time [41]. Thus, from the given input, the proposed MTL-based CNN architecture can effectively identify the bearing health type under inconsistent working conditions, such as conditions with noise, compound faults, and variable motor speeds. Furthermore, a transfer learning (TL)-based framework is adopted to identify bearing faults under certain fault sizes if a pre-trained model (trained on data from a different fault severity condition) is used. Thus, by using a pre-trained deep network and learning parameters from a source task, the proposed framework can mitigate the need for prior knowledge acquisition via training a new model. Therefore, it saves a significant amount of time in the course of task completion [16]. The main contributions of this study can be summarized as follows:

(1) For data preprocessing, a bispectrum-based higher-order analysis is adopted in this work; this can provide distinct patterns under inconsistent working conditions for signals associated with different health conditions. Thus, the inclusion of this signal analysis approach can make the subsequent data classification step easier.
(2) A CNN-based MTL architecture is designed to utilize bispectra as inputs for automatic feature extraction. The end-to-end architecture can predict two types of health characteristics at the same time: (a) the speed of the rotating machine and (b) its health type. For the first time, multitasking capabilities are incorporated into the rolling bearing fault diagnostic architecture.
(3) A transfer learning (TL) framework is incorporated to enhance the classification performance under multiple crack severity conditions. The TL diminishes the need for adjusting CNN architectures with different parameters for inconsistent working conditions.

The proposed approach is tested on two different datasets to validate the generalization ability of the proposed diagnostic framework. Several comparisons with state-of-algorithms are conducted, as well. The rest of the paper is organized as follows. Section 2 talks about the technical backgrounds for the bispectrum, CNN, MTL, and TL-based learning structure and Section 3 introduces the proposed methodology in a detailed, step-by-step procedure. Section 4 shows the experimental details with the performance analysis for different case studies, and Section 5 concludes the paper.

## 2. Technical Background

This section discusses the technical background of the bispectrum and convolutional neural network, as well as the basics of fine-tuning-based transfer learning and multitask learning, which are the essentials of the proposed diagnostic framework.

### 2.1. Bispectrum

The vibration signals of rotating machines contain bearing information with added noise from the surroundings [42]. The addition of ample noise introduces non-stationary, non-linear, and complex behavior into the vibration signals. Thus, it is difficult to capture changes in the frequency over time based on statistical analysis of the signals in the time or frequency domains [43]. Furthermore, due to the non-linear behavior of these signals [1], it is often difficult to extract consistent information from variable working conditions, e.g., variable speeds and multiple crack severities [16,42]. Therefore, to tackle these issues caused by significant variations in the frequency and spectrum amplitudes [42,44], higher-order analysis is considered by using the bispectrum of the signals. The bispectrum is mainly a third-order, spectrum-based approach that searches for non-linear interactions from given signals under inconsistent working conditions; this is done by preserving the phase information while eliminating the Gaussian noise [45]. The insights of bispectrum procedures are given below:

(1) First, a discrete signal is needed, which is defined as follows:

(1) $$\left\{x\left(m\right)\right\}=\left\{x^{p}\left(0\right),x^{p}\left(1\right),\ldots ,x^{p}\left(N-1\right)\right\}\;\mathrm{where}\;p=1,\ldots ,P.$$

(2) The discrete coefficients of this signal can be expressed as:

(2) $$X^{\left(p\right)}\left(\lambda \right)=\frac{1}{N}\sum_{m=0}^{N-1}x^{\left(p\right)}\left(m\right)\exp\left(-j\frac{2\pi m\lambda }{N}\right)\;\mathrm{where}\;\lambda =0,1,\ldots ,\frac{N}{2},p=1,\ldots ,P.$$

(3) The third-order autocorrelation coefficients can be calculated as:

(3) $$\beta_{p}\left(\lambda_{1},\lambda_{2}\right)=\frac{1}{\delta_{0}^{2}}\sum_{i_{1}=-L_{1}}^{L_{1}}\sum_{i_{2}=-L_{1}}^{L_{1}}X^{\left(p\right)}\left(\lambda_{1}+i_{1}\right)X^{\left(p\right)}\left(\lambda_{2}+i_{2}\right)X^{\left(p\right)}\left(-\lambda_{1}-\lambda_{2}-i_{1}-i_{2}\right)\;\mathrm{where}\;\delta_{0}=\frac{f_{s}}{M_{0}}\;\mathrm{and}\;N=\left(2L_{1}+1\right)M_{0}$$

(4) Now, the bispectrum of x(0),x(1),L,x(M−1) can be expressed as:

(4) $$Β\left(\omega_{1},\omega_{2}\right)=\frac{1}{P}\sum_{p=1}^{P}\beta_{p}\left(\omega_{1},\omega_{2}\right)\;\mathrm{where}\;\omega_{1}=\frac{2\pi f_{s}}{M_{0}}\lambda_{1}\;\mathrm{and}\;\omega_{2}=\frac{2\pi f_{s}}{M_{0}}\lambda_{2}.$$

From (4), it is shown that this bispectrum is a two-dimensional reorientation of the spectrum of a signal that considers two independent frequency components $\omega_{1},\omega_{2}$ with a period of 2π. The main advantages of using the bispectrum are given below [42,44,46]:

(1) For the symmetric probability density function of a non-zero Gaussian process, bispectrum is likely to be zeros. Thus, along with removing the Gaussian noise, non-Gaussian components are also extracted from the signals.
(2) For the deterministic stationary signals with no asymmetric component, the bispectrum is zero. However, if some different scenarios come into consideration in the harmonic process, e.g., (a) non-linear interactions or (b) constant components, then the bispectrum has a non-zero value.
(3) From the simple power spectral density-based analysis, the phase coupling information cannot be extracted. Due to the advantages of the identification capability for non-linear systems, the bispectrum can capture the phase coupling information.

Therefore, to develop the diagnostic framework for the rolling element in this study, the bispectrum-based preprocessing method is considered.

### 2.2. Convolutional Neural Network (CNN)

With the advantages of automatic feature extraction, the convolution neural network (CNN) represents an advancement over simple, feed-forward neural architectures with several convolutional and pooling layers and few fully connected layers [16,47]. With the advancement in deep learning-based research, lots of evolutions (e.g., dropout [48,49], batch normalization [50], and global pooling [51]) have been added to the basic architecture of CNN to optimize the network properly and solve the overfitting problem. The training process of this CNN can be described based on two phases: (1) forward propagation and (2) backward propagation.

#### 2.2.1. Forward Propagation

**Figure 1:**
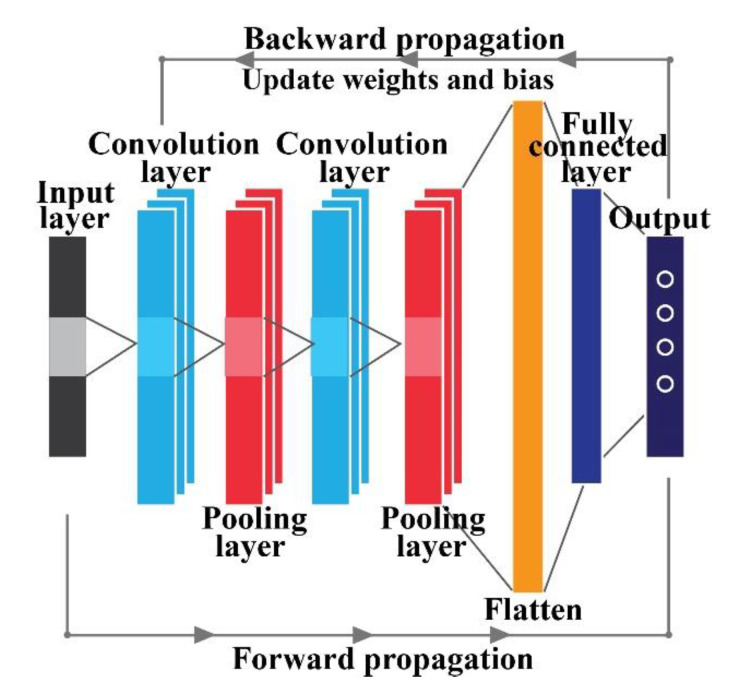
Common architecture of a convolution neural network (CNN).

In the forward propagation phase, the network tries to learn spatial information from the input data during the subsequent layers, as described in Figure 1. Typically, the convolution layer tries to learn abstract features by using kernels of different sizes [43]. To enhance the extracted convolution feature information, a few elements are considered (e.g., the weight, bias, and activation function) with the convoluted features to calculate the final outputs for the next layer [52]. To mitigate the overfitting problem by reducing the redundant features extracted from the convolution layer, a pooling layer is placed immediately after that convolution layer. In this study, by considering the maximum value of the convolutional outputs, max-pooling is used to reduce the size of the learned parameters [53]. Thus, to increase the depth of the network architecture for better learning of the parameters, several convolutions and pooling layers are stacked together. Finally, the outputs of these layers are flattened and connected with some fully connected layers, which alter the resultant matrix into columns [54,55]. The last fully connected layer is denoted as the output layer, from which the output probability is obtained by using activation functions. In this study, SoftMax is considered as the activation function [54]. The SoftMax function is a generalization of the logistic function, which simply squashes values into a given range. Thus, for the simplistic algorithmic approach, it is very fast to train and predict. There are no standard rules for selecting the number of layers of the neural network architecture. From the literature and based on existing knowledge [56,57], it has been shown that the performance increases with a deeper architecture [16]. However, with an increasing number of layers, handling the number of learning parameters is a challenging task to tackle due to the computational complexity [58].

#### 2.2.2. Backward Propagation

Once the forward propagation is finished, the value of the objective function is observed to minimize the loss of the network. Conventionally, the objective function is known as the loss function. The main goal of the neural network-based architectures is to update the parameters of the internal layers by observing and minimizing the objective function. To train the network, the whole dataset is divided into smaller portions in a random manner; this is known as a batch [59]. The size of a batch is dependent on the experimental setup and the length of the total dataset. Thus, to feed the whole dataset into the network for one-time, multiple batches are required. Feeding the whole dataset into the network one time is known as an epoch [59]. By adjusting the bias-variance tradeoff to avoid overfitting and underfitting problems, several epochs are considered to allow the network to enhance the training performance. In this study, the cross-entropy loss function is considered as the objective function to minimize the loss of the target and the actual output [52]. It is a better measure than mean squared error (MSE) for classification because the decision boundary in a classification task is large (in comparison with regression). Moreover, from a probabilistic point of view, the cross-entropy arises as the natural cost function to use as we have SoftMax nonlinearity in the output layer of the network architecture, and we want to maximize the likelihood of classifying the input data correctly. This function can be expressed as follows:

(5) $$L=\frac{1}{n}\sum_{w=1}^{n}\left[y_{w}\ln\bar{y_{w}}+(1-y_{w})\ln\left(1-\bar{y_{w}}\right)\right]$$

Here, *y_w_* and $\bar{y_{w}}$ are the actual target and predictive value of the network in accordance with *w*, respectively.

### 2.3. Multitask Learning with CNN

Multitask learning (ML) is a special type of transfer learning (TL), which follows the main principles of TL by sharing knowledge among the subtasks of a principle task [60,61]. If a task is divided into subtasks, instead of having a separate model for completing each task separately, MTL allows the CNN architecture to create a task branch to perform several subtasks simultaneously by minimizing the one principle objective function [62]. Thus, it shares the model architecture and parameters of the network with all the subtasks, thereby reducing the training time [60]. The concept of MTL can be expressed by the following equations:

(6) $${\left\{x_{m},y_{m}\right\}}_{m=1}^{M},\mathrm{where}\;\left\{ \begin{array}{l} x_{m}=\left\{x_{1}^{m},\ldots ,x_{p}^{m}\right\}\\ y_{m}=\left\{y_{1}^{m},\ldots ,y_{p}^{m}\right\} \end{array}\right.$$

(7) $$y_{n}^{m}=f_{m}\left(x_{n}^{m}\right)$$

**Figure 2:**
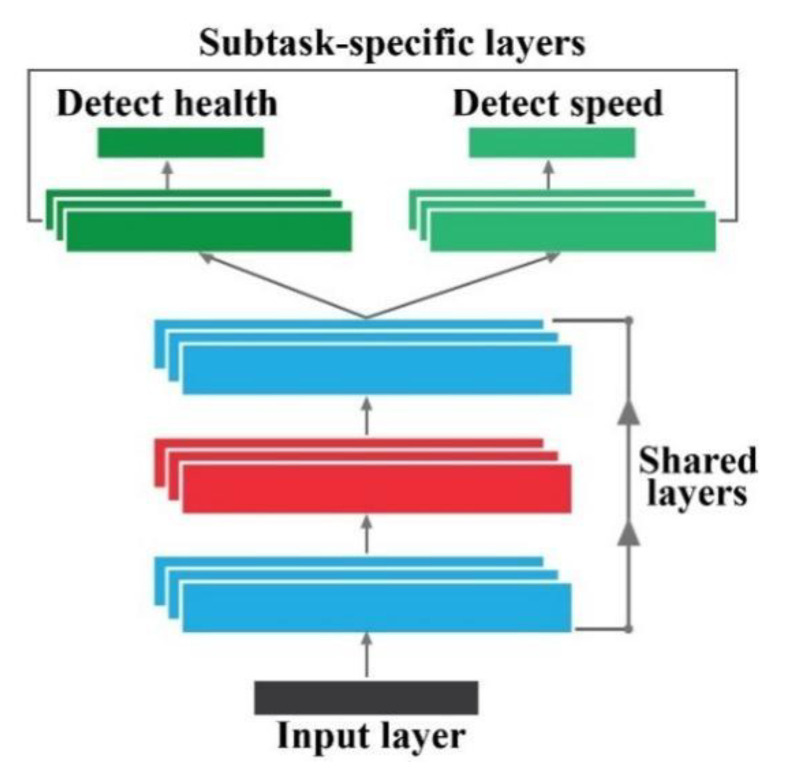
A general framework of a multitask learning neural network.

In (6), ${\left\{x_{m},y_{m}\right\}}_{m=1}^{M}$ refers to p training samples from the original task *M*, where the subscript *m* denotes the subtasks. The aim is to build a CNN-based diagnostic framework for multiple tasks $y_{n}^{m}$ to learn and share transferable parameters to connect different subtasks competently and actively. In Figure 2, the basic idea of MTL is illustrated for visual understanding. In this study, to diagnose different health types along with the speed condition of the bearing, the MTL-CNN is proposed. In the proposed MTL-CNN framework, subtask 1 identifies the rotational speed of the bearing, and subtask 2 identifies the health type of the bearing.

### 2.4. Fine-Tuning-Based Transfer Learning

According to the definition, the main goal of TL is to transfer knowledge obtained from one task to another task that resembles it closely to improve the diagnostic performance of the new task in a short amount of time [13]. Fine-tuning-based TL (FTL) is one of the most popular approaches for designing a TL-based architecture [63]. In FTL, knowledge of the source task is transferred to the target task by transferring the learned parameters only. The source task is the task on which the proposed MTL-CNN is trained, and the parameters are adjusted accordingly. Alternatively, the target task is a task that is very similar to the source task; the learned parameters can be transferred from the source task to the target task to make the training process faster [16]. In this study, for the source task, the MTL-CNN is trained to identify the bearing speed conditions and health type under a certain crack severity. Then, in the target task, this learned knowledge and the network parameters are passed to identify the speed conditions and health type of the bearing for a different crack severity. Thus, the necessity of training the target task from scratch is mitigated. For example, (7) can be rewritten as the output of the source task:

(8) $$S_{y_{n}^{m}}=S_{f_{m}}\left(S_{x_{n}^{m}}\right)$$

where $S_{f_{m}}$ is the final objective function or mapping function of the source task. Similarly, like the source task, the relatively similar target task can be expressed as:

(9) $$T_{y_{n}^{m}}=T_{f_{m}}\left(T_{x_{n}^{m}}\right)$$

where $T_{f_{m}}$ is the final objective function of the target task. In the FTL framework, by using the MTL-CNN architecture, the network first learns the mapping function $S_{f_{m}}$. After that, $S_{f_{m}}$ is transferred to $T_{y_{n}^{m}}$ for obtaining the optimized objective function $T_{f_{m}}$, which improves the learning process. In this study, FTL is used for inter-task diagnostic purposes.

## 3. Proposed Methodology

**Figure 3:**
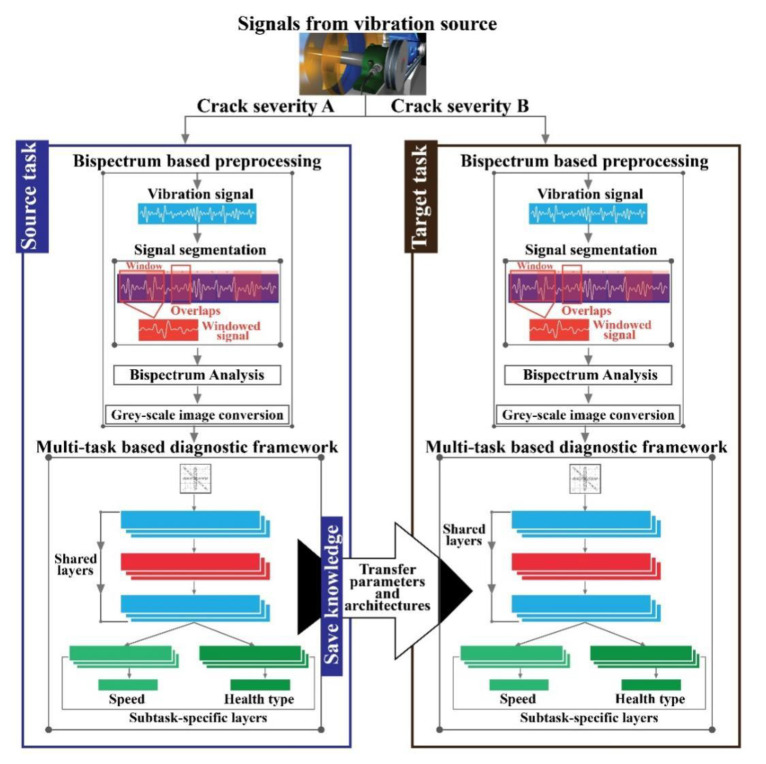
The block diagram of the proposed diagnostic method.

The main objective of this research is to identify the health states of rolling element bearings by using bispectra analysis of the vibration signals and MTL-based transfer learning under inconsistent working conditions. Figure 3 illustrates the block diagram of the proposed methodology in detail.

As can be seen from the figure, the proposed framework is composed of three core steps:

(1) *Source task*: The testbed vibration signals associated with a certain crack severity under variable speed conditions are converted to a 2D bispectrum for deep learning-based analysis. The proposed MTL-CNN identifies the health type and the speed conditions of the preprocessed bispectrum of a certain crack severity. Thus, in the source task, the proposed MTL-CNN framework learns invariant spatial information for inconsistent working conditions that can optimize the developed network architecture.
(2) *Transfer pool*: The knowledge obtained by an optimized network, also referred to as the transfer pool in the source task, is then passed to the target task. The benefits of the transfer pool are: (a) the knowledge transferred between the tasks can work as prior information to the target task, which can boost its diagnostic performance, and (b) the need to train the whole network to complete targets diminishes. Therefore, the overall learning process becomes faster if unseen data associated with a different crack size and motor speed is experienced in the target task.
(3) *Target task*: In the target task, data associated with different crack severities (different than the source task) under variable speed conditions are passed to the diagnostic framework. Like the source task, 2D bispectra of the vibration signals are computed here, as well. Then, to identify the speed conditions and the health type, the trained network from the source task (with the parameters and learned knowledge) is utilized. Both source and target tasks have similarities in terms of the diagnostic framework design.

### 3.1. Bispectrum

For a neural network-based diagnostic framework, data preprocessing is the most crucial part due to the large volume of the dataset [64]. Additionally, a significant amount of time is required due to the calculation of multiple features associated with inconsistent working conditions. Therefore, to solve these issues, 2D bispectrum-based analysis is considered in this study. As can be seen from Figure 3, the 1D vibration signals are first segmented into smaller portions with a length of 2048 data points based on the overlapping technique. Next, a bispectrum with dimensions of 128×128 is computed for each segment. Finally, these 2D bispectra are converted into grey-scale images for further analysis.

### 3.2. Multitask Learning-Based CNN Architecture

**Figure 4:**
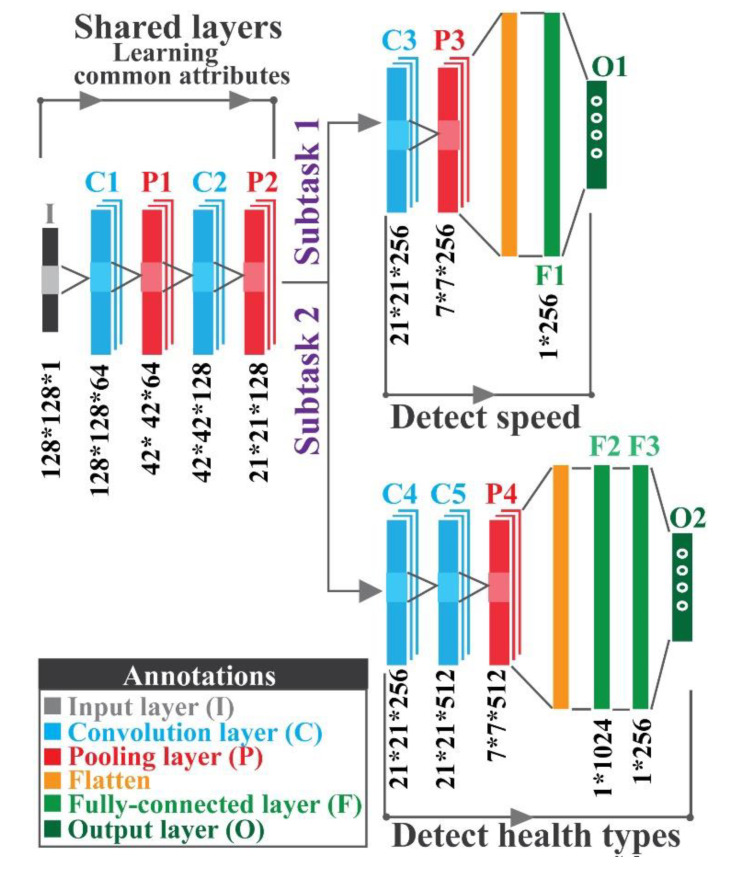
Proposed MTL-CNN architecture with layer-wise specifications.

The proposed MTL–based, inter-class diagnostic framework is developed using a CNN for identifying the health types of bearings under variable speed conditions and multiple fault severities. As presented in Figure 4, the designed architecture of the MTL-CNN can be divided into two segments: (a) a general feature extractor and (b) subtask branches. The general feature extractor takes abstract spatial information from the preprocessed 2D input data. This segment is composed of two convolutions and two pooling layers. Later, the subtask branches utilize the extracted spatial information to identify the health types and the speed conditions of bearings. In the proposed framework, subtask 1 is used to identify the speed of the bearing, which is composed of one convolution layer, one pooling layer, one fully connected layer, and the final output layer. Similarly, subtask 2 is allocated for identifying the health state of the bearing, which is composed of two convolution layers, one pooling layer, two fully connected layers, and the final output layers. For the activation of the fully connected layers of this framework, leaky rectified linear unit (Leaky ReLU) [65] is considered. To prevent the overfitting problem, L2 regularization with a value of 0.04 is attained on the layer before the output layer. By observing the behavior of training accuracy, and loss function values in several experiments, this value has been decided for avoiding the overfitting problem. Similarly, the number of kernels of the proposed MTL-CNN architecture is optimized utilizing grid search, as preliminary experiments demonstrate that the fluctuating number of kernels affects the final classification performance [66].

### 3.3. Fine-Tuned Transfer Learning Framework

In the source task, MTL-CNN is optimized on data that are associated with a single crack size. The learned parameters of the source task are then passed to the target task to identify the faults using a dataset collected from the same testbed with different crack severities. The components of the proposed MTL-CNN with layer-wise (layers are denoted in Figure 4) transferable specifications are presented in Table 1.

### 3.4. Performance Evaluation

To evaluate the performance of the proposed framework, different performance evaluation matrices have been considered in this work, including the (a) F1 score (F1), (b) average score (AS), (c) confusion matrices [67], and (d) loss function graph. The F1 score and AS [68] can be calculated as expressed in Equations (10) and (11), respectively:

(10) $$F1=\frac{2TP}{2TP+FN+FP}\times 100\%$$

(11) $$AS=\frac{\sum F1}{total\_classes}$$

In (10), the terms TP, FN, and FP represent the number of true positives, false negatives, and false positives, respectively. Moreover, to adjust the overfitting and underfitting problems, the total loss of the model is observed until 3000 epochs. Further, to visualize the class separability, the feature space of the output layer is visualized by t-stochastic neighbor embeddings (t-SNEs) [69]. Subsequently, to remove the bias from the data along with the evaluation parameters, six-fold cross-validation (6-CV) [70] is used for each experiment.

## 4. Experimental Set Up and Performance Analysis

In this study, several experiments are performed to verify the efficiency of the diagnostic framework under inconsistent working conditions, including different crack severities, speeds (RPM), load conditions, and low signal-to-noise ratios (SNRs). The proposed approach is validated with two separate datasets, i.e., (a) publicly available bearing dataset provided by Case Western Reserve University (CWRU) [71] and (b) a rolling element bearing dataset with compound faults acquired from a self-designed testbed. The reason for using a public dataset is to validate the generalization capabilities of the proposed diagnostic framework; furthermore, low SNR tests are conducted using only this dataset for easy replication.

### 4.1. Case Study 1: Case Western Reserve University Dataset

#### 4.1.1. Experimental Setup and Dataset Description

**Figure 5:**
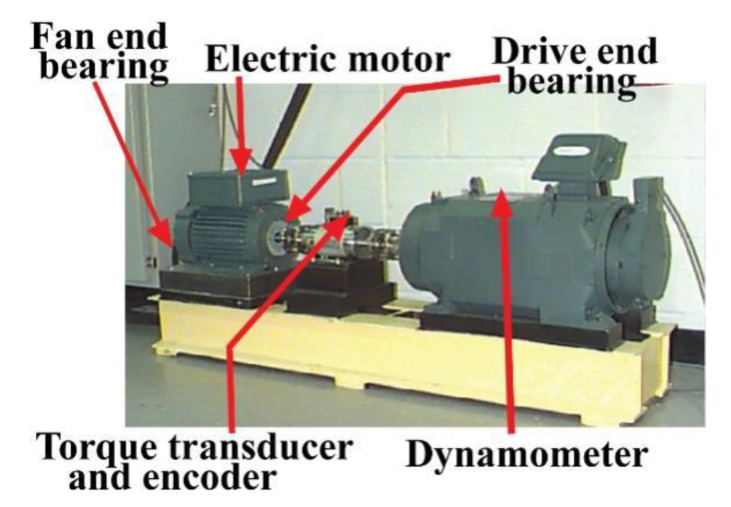
CWRU bearing testbed [71] for collecting vibration signals.

For this experiment, vibration signals from rolling element bearings are collected from a public data repository offered by the bearing data center of Case Western Reserve University (CWRU) [71]. Figure 5 depicts the whole experimental setup. It can be observed that the experimental setup is composed of an induction motor (2 hp), a dynamometer, and a transducer. The signals are collected with the help of accelerometers, which are mounted on the housing of the induction motor. During signal collection, several motor loads were applied by using a dynamometer. As a result, variation in the motor shaft speed was also observed. Furthermore, to create the artificially induced faults on the drive-end bearing, an electromagnetic discharge machine was used. The signals were collected with a sampling frequency of 12 kilohertz (kHz). While conducting the experiments, four types of health conditions of the bearing are considered under different motor speeds and crack severities, i.e., normal type (NT), inner raceway type (IRT), outer raceway type (ORT), and roller type (RT). In total, 800 signals (200 from each health type) are recorded for each motor speed (measured in revolutions per minute, RPM): 1797, 1772, and 1750 RPM. The details of the datasets are given in Table 2.

#### 4.1.2. Performance Analysis

**Figure 6:**
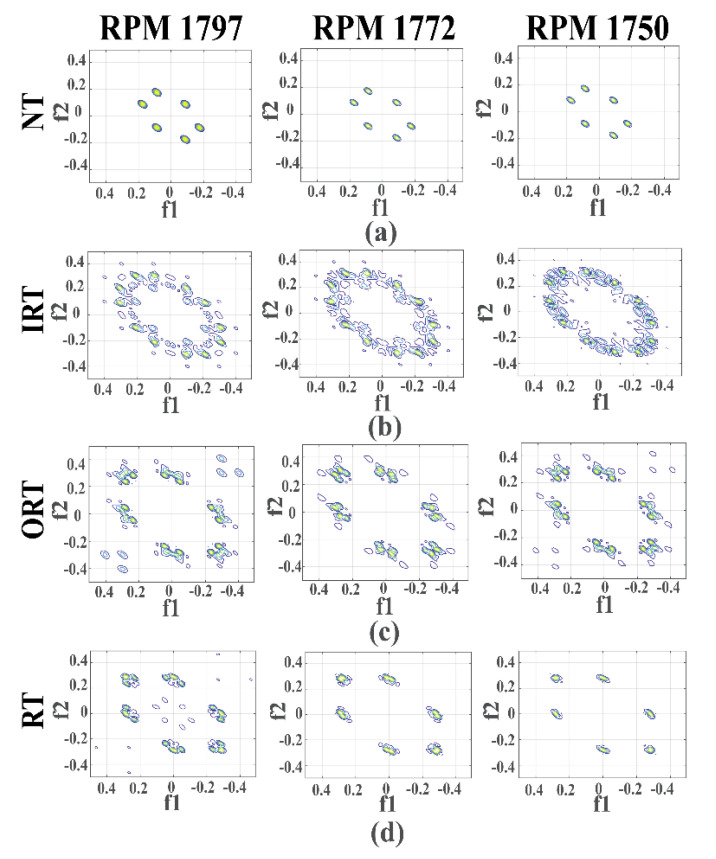
Visualization of the bispectra associated with different health types under various speeds: (**a**) normal type (NT), (**b**) inner raceway type (IRT), (**c**) outer raceway type (ORT), and (**d**) roller type (RT).

Initially, in the source task, the proposed MTL-CNN is trained with one type of crack severity. Later, the learned knowledge is passed to the target task, where the datasets with different crack severities are tested. For both source and target tasks, all the datasets need to be preprocessed by the proposed bispectrum-based method. The bispectra obtained from dataset 1 are presented in Figure 6. As can be seen from the figure, the pattern of the bispectrum for the NT condition is consistent under varying shaft speeds and load conditions. For IRT, the prominent peaks of the bispectrum can be observed at 0.1 Hz, 0.2 Hz, and 0.25 Hz. Subsequently, from the bispectra of ORT and RT, it is observed that the prominent peaks lie between 0 Hz and 0.4 Hz. It should be noted that, in the bispectra for IRT, ORT, and RT, several discreet peaks are visible at different symmetric locations. Due to the presence of additive noise in the original vibration signals, these types of variations are encountered in the bispectra. However, for all the considered health types, the pattern of the dominant peaks is very similar. Thus, the obtained bispectra deliver invariability under variable shaft speeds and load conditions. These bispectra are fed into the proposed MTL-CNN so that it can automatically extract salient information from the images, which can be subsequently used for multi-class classification [16,72]. Once the source task is accomplished, the target task is started to measure the final diagnostic performance. Specifically, in the source task, the MTL-CNN is trained with one dataset. Those MTL-CNN parameters and architectures are then passed to the target task to identify the bearing speeds and health types for rest of the datasets. The parameters of the MTL-CNN architecture are depicted in Figure 4.

To generate results, a total of three experiments are conducted. In experiment 1, dataset 1 is used for the source task, while datasets 2 and 3 are considered in the target task. At first, the bispectrum images are attained from dataset 1, and then the MTL-CNN is trained and tested with 90% and 10% of the data, respectively. The trained MTL-CNN architecture with learned weights is then saved to use for the target task. In the target task, the bispectrum-based inputs are calculated from datasets 2 and 3. Then, the MTL-CNN architecture with the learned knowledge is used to adjust the target task’s MTL-CNN for measuring the final diagnostic performance. In this case, 15% of data from datasets 2 and 3 are used for training the network, and the remaining 85% of the data from both datasets are used for testing purposes. Similarly, for experiment 2, dataset 2 is considered for the source task and datasets 1 and 3 are considered for the target task. For experiment 3, dataset 3 is considered for the source task and datasets 1 and 2 are considered for the target task. Therefore, in this diagnostic framework, the dataset is divided separately for two different tasks, i.e., the source task and the target task, as shown in Table 3.

**Figure 7:**
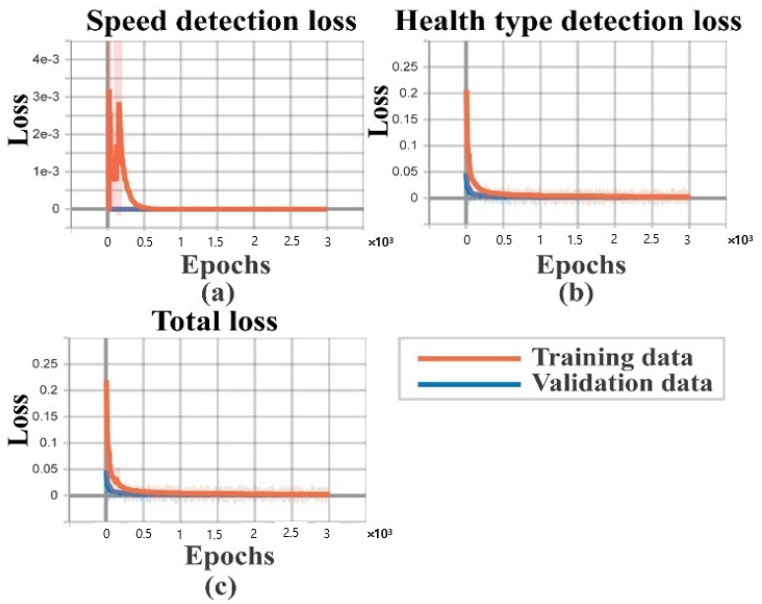
Loss functions for the target task of experiment 3: (**a**) training and validation loss for task A: speed detection, (**b**) training and validation loss for task B: health type detection, and (**c**) training and validation loss for the TL-based MTL-CNN model.

**Figure 8:**
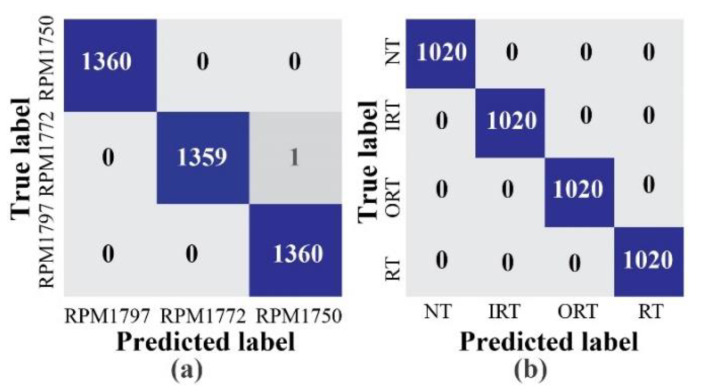
Confusion matrices for the target task of experiment 3: (**a**) task A: speed detection and (**b**) task B: health type detection.

**Figure 9:**
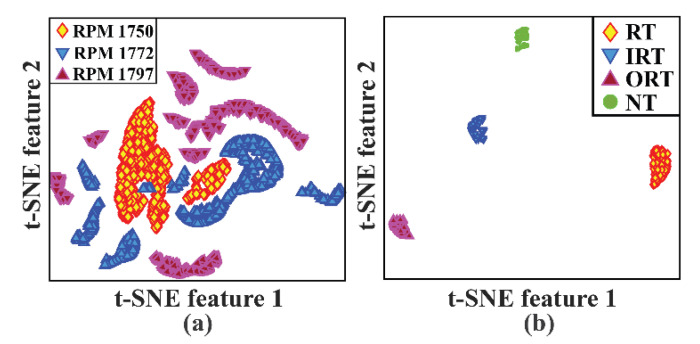
t-SNE features of the output layers for the target task of experiment 3: (**a**) task A: speed detection and (**b**) task B: health type detection.

Additionally, to remove the bias from the datasets, an equivalent number of samples are considered for each health type. Moreover, the model is trained for 3000 epochs for the source tasks. For each experiment, once the network is trained in the source task, the performance of the target task is observed to measure the final classification accuracy. The diagnostic performances of the three experiments are listed in Table 4. The obtained results in terms of F1 and AS are calculated by Equations (9) and (10). As can be seen from this Table, the subtasks for all three experiments, apart from subtask B for experiment 3, achieved 100% accuracy. In experiment 3, the source dataset is dataset 3, which contains a crack severity of 0.021. In this case, the speed detection accuracy for 1772 RPM data in the target task is 99.9%. To show the detailed analysis of this specific result, the graph of the overall loss functions, as well as the subtask-specific loss functions, are highlighted in Figure 7. Additionally, the confusion matrices for these two subtasks, along with the last layer feature separability obtained by t-SNE, are highlighted in Figure 8 and Figure 9. As expected, from Figure 9, the two-dimensional t-SNE features are of high quality that the trained network architecture can estimate both the intraclass compactness and interclass separability for both the subtasks (Task A, and task B).

**Figure 10:**
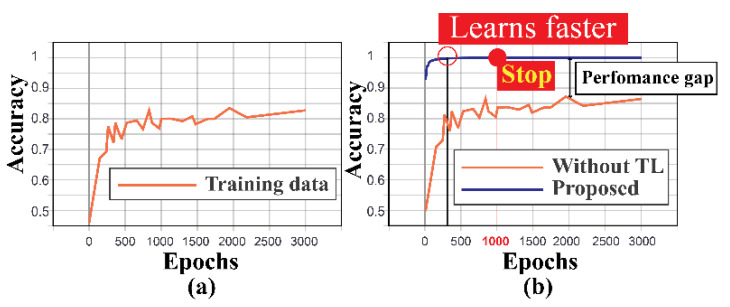
(**a**) The training accuracy typically achieved with dataset 1 and 2 for experiment 3: target task and (**b**) comparison of the training accuracies for the two approaches (without TL vs. the proposed approach).

As mentioned earlier, the TL-based approach can learn faster with a smaller amount of data. To establish this fact, experiment 3 is further analyzed. A test is conducted where the proposed MTL-CNN is trained from scratch with the target dataset independently, without passing on any knowledge from the source task. Like the previous test, 15% of the data are used for training and 85% of the data are used for testing the network. The data division is identical so that the performance can be compared on the same scale. In Figure 10a, it can be observed that the MTL-CNN without TL does not yield the same performance; it provides 83.3% accuracy for overall training. Furthermore, from Figure 10b, it is observed that the proposed approach learns faster than the MTL-CNN without the TL approach, and it also achieved 100% training accuracies, which lead to a better diagnostic performance (as discussed in Table 4). Without TL, it took around 3000 epochs to train the network, whereas, with TL, with 1000 epochs, better performance is achieved. It makes the training process faster by 3× times. Hence, it can be concluded that the proposed model provides a faster convergence rate with enhanced classification performance.

To establish the validity and robustness of the proposed diagnostic framework, several approaches are considered from the literature [43,73,74] and adopted using an experimental setup similar to the one in this case-study. The AS accuracy is considered to compare the performances of these methods. These methods include:

(1) WC + MTL-CNN-TL: the input is transformed into 2D wavelet coefficient matrices and then supplied to the MTL-based deep architectures [73] to perform the TL-based analysis on the target task.
(2) TFI + CNN-TL: the input is transformed into several time-frequency images (TFI) to create the multi-fusion input [43] and then passed to the MTL-CNN architecture based on the CNN model mentioned in [43]. Finally, the TL-based approach from the proposed framework is adopted to perform the final analysis.
(3) S-transform + CNN-TL: the input is transformed into 2D vibration images by using Stockwell transform [13] and then supplied to the CNN-based deep architectures mentioned in [13] to perform the TL-based analysis on the target task.
(4) RAW + CNN-TL: the input is directly fed to the adopted CNN architecture derived from [74], and then the knowledge attained from the source task is transferred to the target task to determine for final classification accuracy.

**Figure 11:**
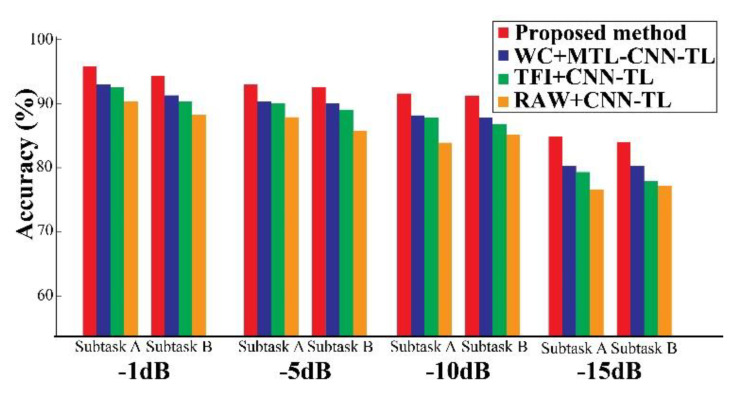
Impact of noisy data on classification performance for the target task of experiment 3.

These methods are compared, and the improvement details of the proposed framework are discussed in Table 5. It is clear that the proposed framework outperforms these other state-of-the-art approaches [43,73,74], showing an improvement of 3.7% to 9.0% in terms of the AS score. Additionally, the impact of noisy data on the diagnostic performance of the proposed approach is also explored. Experiment 3 is again considered for this test. The source task is trained with dataset 3. Then, Gaussian white noise (AWGN) with signal to noise ratios (SNRs) ranging from −1 dB to −15 dB is added to the target datasets, i.e., datasets 2 and 3, for validating the diagnostic performance. As can be seen from Figure 11, the diagnostic performance in this scenario is declined slightly. Especially, for a high level of AWGN (i.e., for −15 dB), the performance dropped off, and the classification accuracy is around 85%. However, the proposed method still performs better in noisy conditions than the other methods used for comparison. Therefore, these results show that the proposed diagnostic framework can tolerate moderate AWGN and yield acceptable diagnostic performance.

### 4.2. Case Study 2: Self-Designed Test Rig with Compound Faults

#### 4.2.1. Experimental Setup and Dataset Description

**Figure 12:**
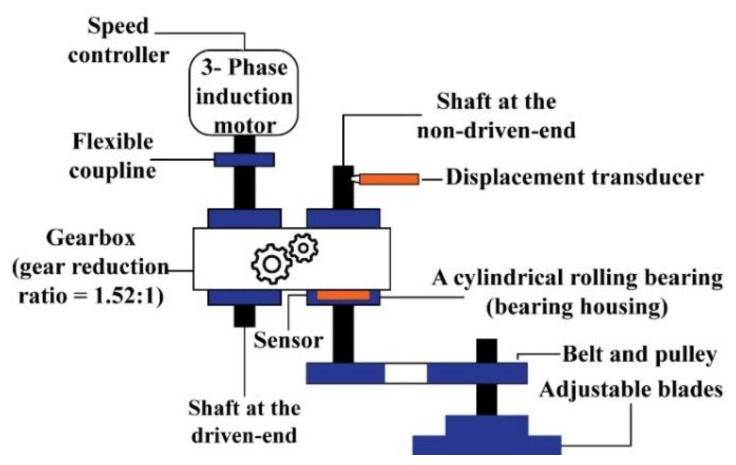
Schematic representation of the self-designed testbed.

To collect data with compound fault conditions, a test on a self-designed test rig is conducted. The vibration signals are collected at two different motor speeds, i.e., 300 and 400 RPM, for two different crack severities. The experimental setup is illustrated in Figure 12. As depicted in the figure, the setup uses two shafts, i.e., drive-end and non-drive-end shafts. These two shafts are connected to a gearbox that has a reduction ratio of 1.52:1. Furthermore, a cylindrical bearing is used (model FAG-NJ206-E-TVP2) at both shaft ends. To obtain the data, a three-phase induction motor is positioned in the drive-end shaft [42]. A wide-band vibration sensor [75] is applied to record the vibration signals from the non-drive-end shaft at a sampling rate of 65,536 Hz [42].

**Figure 13:**
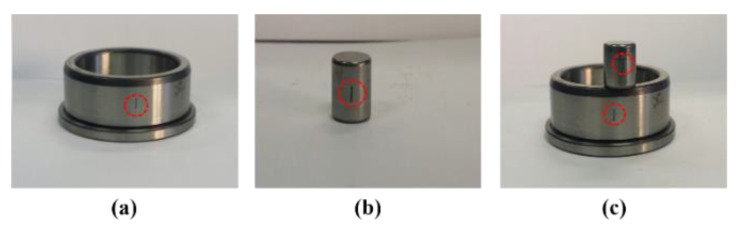
Fault types: (**a**) inner raceway type (IRT), (**b**) roller type (RT), and (**c**) inner-roller type (IART).

In the experiment, among the four types of health conditions as shown in Figure 13, one compound fault is considered, i.e., normal type (NT), inner raceway type (IRT), roller type (RT), or inner-roller type (IART). A total of 800 signals (200 from each health type) are recorded at each of the considered motor speeds (300 and 400 RPM). Details of the datasets are listed in Table 6.

#### 4.2.2. Performance Analysis

**Figure 14:**
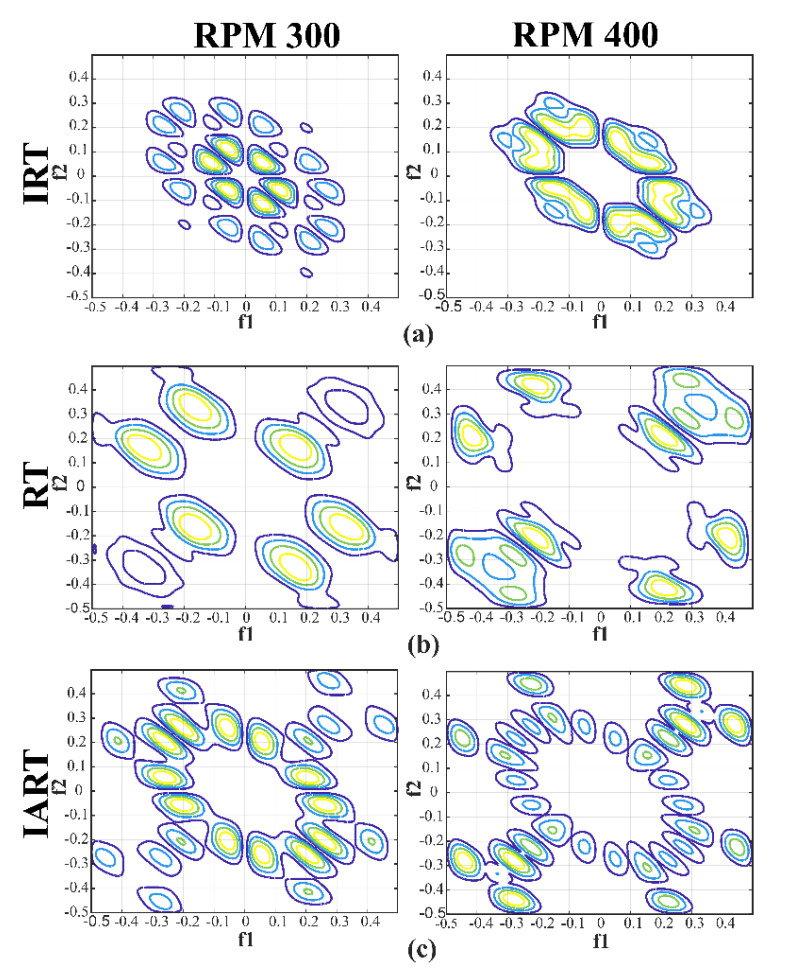
Bispectrum visualization of different health types across various speeds: (**a**) inner raceway type (IRT), (**b**) roller type (RT), and (**c**) inner-roller type.

To perform a diagnostic analysis, two experiments are conducted. In experiment 1, dataset 1 is used for the source task and dataset 2 is used for the target task. In experiment 2, dataset 2 is utilized for the source task and dataset 1 is used for the target task. For these two experiments, the datasets are divided into the training and testing subsets, as shown in Table 7. The bispectra are calculated from the samples of both datasets, i.e., datasets associated with the source and target tasks. As can be seen in Figure 14, the computed bispectra from dataset 1 for RT and IART under different RPMs are similar. However, there is a slight variation in the pattern of the bispectra for IRT. Nevertheless, the prominent peaks are between 0.02 and 0.2 Hz. Furthermore, for RT, the prominent peaks lie between 0.14 Hz and 0.47 Hz. Therefore, the prominent peaks of IART (compound fault conditions) should consider the ranges of both IRT and RT conditions. From Figure 14, the prominent peaks of IART fall between 0.02 Hz and 0.5 Hz. Thus, along with the visual similarities in the patterns of different RPM conditions, the frequency range of significant peaks suggests that IART preserves the characteristics of both IRT and RT together.

After that, for each experiment, the diagnostic performance of the target task is attained. The diagnostic performance of the proposed approach is listed in Table 8. The experimental details are kept identical to the previous case study. In Table 8, the results indicate that, for speed detection, the proposed framework performs as expected, like the previous case study. However, for health type detection, the performance (AS) deteriorated slightly in both experiments. It is evident from the obtained results that the framework fails to give 100% accuracy. Nonetheless, the experimental results prove that the proposed TL-based MTL-CNN framework can still robustly identify the health conditions of a rolling element bearing under variable load, speed, and compound fault conditions.

## 5. Conclusions

This study presented a bispectrum-aided preprocessing technique, which is combined with a multitask learning–based, fine-tuned transfer learning approach to diagnose bearing faults. Multitask learning is implemented by using a convolutional neural network. This represents a new diagnostic approach for rolling element bearings operating under inconsistent working conditions, e.g., different speeds, different loads, and various crack severities. Moreover, the proposed architecture is also tested with a bearing dataset containing ample noise. Bispectra of the vibration signals under inconsistent working conditions portray a distinct pattern that enhances the performance of the subsequent classification step. By integrating the multitask learning ability of deep architectures with transfer learning-based automatic feature analysis, this method can explore abstract features from a higher-order spectrum for tackling non-stationary, non-linear, and noisy vibration signals. The adaptation of this approach enables end-to-end diagnosis without requiring any statistical feature extraction or selection procedure for variable working conditions. Data acquired from two sources, i.e., (a) a publicly available standard dataset from Case Western Reserve University’s archive and (b) a self-designed testbed, are used to validate the robustness and the generalization capabilities of the proposed approach. Experimental results suggest that this approach can enhance the diagnostic performance, while also saving a lot of time. This approach makes use of bispectra with a fixed resolution, which could be replaced with bispectra that have an adaptive resolution to better address the issues faced during the fault diagnosis of nearing due to the inconsistent working conditions of a rotating machine. Furthermore, an unsupervised multitask deep network could be utilized to enhance the generalization ability of the proposed diagnostic framework.

## Figures and Tables

**Table 1:** The details transferred architecture to the target task using the proposed CNN-MTL.

Subtask 1			Subtask 2		
Layers	Trainable	Transfer	Layers	Trainable	Transfer
C1	Yes	Yes	C4	Yes	Yes
P1	No	Yes	C5	Yes	Yes
C2	Yes	Yes	P4	No	Yes
P2	No	Yes	F2	Yes	No
C3	Yes	Yes	F3	Yes	No
P3	No	Yes	O2	Output	No
F1	Yes	No			
O1	Output	No			

**Table 2:** Details of CWRU dataset used for this experiment.

	Health Type	Shaft Speed (RPM)	Load	Crack Size
				Length (in)
Dataset 1	NT	1797, 1772, 1750	0, 1, 2	-
	IRT			0.007
	ORT			0.007
	RT			0.007
Dataset 2	NT	1797, 1772, 1750	0, 1, 2	-
	IRT			0.014
	ORT			0.014
	RT			0.014
Dataset 3	NT	1797, 1772, 1750	0, 1, 2	-
	IRT			0.021
	ORT			0.021
	RT			0.021

**Table 3:** Data division.

**Source task details**	**Dataset**	**Train (90%)**		**Test (10%)**
		**Training (80%)**	**Validation (20%)**	
	1	1944 samples	216 samples	240 samples
	2	1944 samples	216 samples	240 samples
	3	1944 samples	216 samples	240 samples
**Target task details**		**Train (15%)**		**Test (85%)**
		**Training (90%)**	**Validation (10%)**	
	1	324 samples	36 samples	2040 samples
	2	324 samples	36 samples	2040 samples
	3	324 samples	36 samples	2040 samples

**Table 4:** Diagnostic performance of case study 1.

Exp.	Source Task	Target Task	Subtasks	Conditions	F1 (%)	AS (%)
1	Dataset 1	Dataset 2, 3	A. Speed detection	1797 RPM	100	100
				1772 RPM	100	
				1750 RPM	100	
			B. Health type detection	NT	100	100
				IRT	100	
				ORT	100	
				RT	100	
2	Dataset 2	Dataset 3, 1	A. Speed detection	1797 RPM	100	100
				1772 RPM	100	
				1750 RPM	100	
			B. Health type detection	NT	100	100
				IRT	100	
				ORT	100	
				RT	100	
3	Dataset 3	Dataset 1, 2	A. Speed detection	1797 RPM	100	99.9
				1772 RPM	99.9	
				1750 RPM	100	
			B. Health type detection	NT	100	100
				IRT	100	
				ORT	100	
				RT	100	

**Table 5:** Comparison of the diagnostic performance for case study 1.

Methods	Exp.	Subtasks	AS (%)	Improvement Shown by the Proposed Approach (%)
WC + MTL-CNN-TL	1	A	96.2	3.8
		B	96.3	3.7
	2	A	96.3	3.7
		B	95.9	4.1
	3	A	96.1	3.9
		B	96.2	3.8
TFI + CNN-TL	1	A	97.2	2.8
		B	96.5	3.5
	2	A	96.5	3.5
		B	96.9	3.1
	3	A	95.2	4.7
		B	96.3	3.7
S-transform + CNN-TL	1	A	99.2	0.8
		B	98.1	1.9
	2	A	99.5	0.5
		B	99.1	0.9
	3	A	99.0	0.9
		B	98.7	1.3
RAW + CNN-TL	1	A	92.1	7.9
		B	92.3	7.7
	2	A	92.0	8.0
		B	91.9	8.1
	3	A	90.9	9.0
		B	91.2	8.8
Proposed	1	A	100.0	-
		B	100.0	-
	2	A	100.0	-
		B	100.0	-
	3	A	99.9	-
		B	100.0	-

**Table 6:** Details of the working conditions.

	Health Type	Shaft Speed (RPM)	Crack Size
			Length (mm)
Dataset 1	NT	300, 400	-
	IRT		6
	RT		6
	IART		6
Dataset 2	NT	300, 400	-
	IRT		12
	RT		12
	IART		12

**Table 7:** Data division.

**Source task details**	**Dataset**	**Train (90%)**		**Test (10%)**
		**Training (80%)**	**Validation (20%)**	
	1	1152 samples	288 samples	160 samples
	2	1152 samples	288 samples	160 samples
**Target task details**		**Train (15%**)		**Test (85%)**
		**Training (90%)**	**Validation (10%)**	
	1	216 samples	24 samples	1360 samples
	2	216 samples	24 samples	1360 samples

**Table 8:** Diagnostic performance for case study 2.

Exp.	Source Task	Target Task	Subtasks	Conditions	F1 (%)	AS (%)
1	Dataset 1	Dataset 2	A. Speed detection	300 RPM	98.2	98.2
				400 RPM	98.1	
			B. Health type detection	NT	95.2	94.8
				IRT	93.4	
				RT	96.1	
				IART	94.3	
2	Dataset 2	Dataset 1	A. Speed detection	300 RPM	98.4	98.3
				400 RPM	98.2	
			B. Health type detection	NT	96.1	95.1
				IRT	95.2	
				RT	95.5	
				IART	93.5	
